# De l'Agrandissement Momentané du Bassin

**Published:** 1895-03

**Authors:** 


*De l'Agrandissement Momentané du Bassin*. Par ADOLPHE PINARD. Pp. 59. Paris: G. STEINHEIL. 1894.

For the last few years M. Pinard has been a warm advocate for the operation of symphysiotomy, and the present excellent monograph shows that a wider experience has only confirmed his earlier views on the subject.

The work is divided into the following sections: (1) Clinical and statistical. (2) The anatomy and physiology of the sacro-iliac articulations before and after puberty. (3) Mensuration and indications for symphysiotomy. (4) The operative technique. The results at the Baudelocque clinique have been so good that they compare favourably with the operation for induction of premature labour; thus, during a portion of 1892-1894 there were thirty-six symphysiotomies (twenty-three of these being performed by the author), one pubiotomy and one ischio-pubiotomy (Farabeuf's operation). Of these thirty-eight cases, thirty-six women recovered and two died; and thirty-four children were born alive and four dead: whereas, of sixty-four cases of induction of premature labour, sixty-two women recovered and two died; and thirty-five children only were born living and thirty dead.

The work is carefully written, and the subject scientifically considered. There are several useful plates and diagrams, and the book has been printed and prepared in that clear way which makes so many of the Continental *brochures* pleasant to read. It is an excellent addition to the literature of the subject.

